# Physical Examinations via Video for Patients With Heart Failure: Qualitative Study Using Conversation Analysis

**DOI:** 10.2196/16694

**Published:** 2020-02-20

**Authors:** Lucas Martinus Seuren, Joseph Wherton, Trisha Greenhalgh, Deborah Cameron, Christine A'Court, Sara E Shaw

**Affiliations:** 1 Nuffield Department of Primary Care Health Sciences University of Oxford Oxford United Kingdom; 2 Faculty of Linguistics University of Oxford Oxford United Kingdom

**Keywords:** remote consultation, telemedicine, videoconferencing, communication, language, linguistics, gestures, physical examination

## Abstract

**Background:**

Video consultations are increasingly seen as a possible replacement for face-to-face consultations. Direct physical examination of the patient is impossible; however, a limited examination may be undertaken via video (eg, using visual signals or asking a patient to press their lower legs and assess fluid retention). Little is currently known about what such video examinations involve.

**Objective:**

This study aimed to explore the opportunities and challenges of remote physical examination of patients with heart failure using video-mediated communication technology.

**Methods:**

We conducted a microanalysis of video examinations using conversation analysis (CA), an established approach for studying the details of communication and interaction. In all, seven video consultations (using FaceTime) between patients with heart failure and their community-based specialist nurses were video recorded with consent. We used CA to identify the challenges of remote physical examination over video and the verbal and nonverbal communication strategies used to address them.

**Results:**

Apart from a general visual overview, remote physical examination in patients with heart failure was restricted to assessing fluid retention (by the patient or relative feeling for leg edema), blood pressure with pulse rate and rhythm (using a self-inflating blood pressure monitor incorporating an irregular heartbeat indicator and put on by the patient or relative), and oxygen saturation (using a finger clip device). In all seven cases, one or more of these examinations were accomplished via video, generating accurate biometric data for assessment by the clinician. However, video examinations proved challenging for all involved. Participants (patients, clinicians, and, sometimes, relatives) needed to collaboratively negotiate three recurrent challenges: (1) adequate design of instructions to guide video examinations (with nurses required to explain tasks using lay language and to check instructions were followed), (2) accommodation of the patient’s desire for autonomy (on the part of nurses and relatives) in light of opportunities for involvement in their own physical assessment, and (3) doing the physical examination while simultaneously making it visible to the nurse (with patients and relatives needing adequate technological knowledge to operate a device and make the examination visible to the nurse as well as basic biomedical knowledge to follow nurses’ instructions). Nurses remained responsible for making a clinical judgment of the adequacy of the examination and the trustworthiness of the data. In sum, despite significant challenges, selected participants in heart failure consultations managed to successfully complete video examinations.

**Conclusions:**

Video examinations are possible in the context of heart failure services. However, they are limited, time consuming, and challenging for all involved. Guidance and training are needed to support rollout of this new service model, along with research to understand if the challenges identified are relevant to different patients and conditions and how they can be successfully negotiated.

## Introduction

### Background

Video consultations using technology such as FaceTime (Apple Inc) offer potential benefits to patients (eg, increased access) [1-4] and health services (eg, improved efficiency of care) [5]. There has been a significant push by policymakers to develop video consultation services [6-8]. Clinicians and patients are receptive, particularly with regard to the management of long-term conditions [1]. However, uptake has been limited to date [9].

In video consultations, patients and clinicians have no shared physical environment [10]. This makes direct physical examination impossible (eg, using touch to palpate parts of the body) and places limits on uptake. In theory, a *video examination* is possible (eg, using vision to assess a patient’s skin color or guiding a relative to use a blood pressure monitor). Studies on, for example, chronic obstructive pulmonary disease [11] and asthma [12], show that patients can use technology to monitor their own condition.

Little is currently known about when it is (and is not) possible to conduct a video examination. Clinicians and patients appear cautious [13], with video examinations frequently regarded as problematic [14], and patients requiring physical examinations often excluded from studies [15,16]. The little evidence that is available suggests that it is possible, in some cases, to conduct examinations remotely. One qualitative study of consultations using a telephone helpline in Australia found that nurses could guide patients to do their own examinations by giving simple instructions and asking patients about the *normality* of what they saw and felt [17]. Another study of remote play–based therapy enabled clinicians (at one end) to use toys to interact positively with young children (at the other end) [18]. Finally, a study of televascular consultations showed that specialists (in the clinic) could collaborate effectively with nurses (with the patient) to aim a camera, manipulate the patient’s body, and provide assessments [19]. Caution is, however, required. One study on teledermatology showed that although skin lesions can be assessed over video, even high-resolution images cannot completely replace in-person assessment [20].

### Objectives

Current research suggests that video examinations may be possible. However, questions remain about how they might be accomplished in practice and with which patients and conditions. Participants have to accomplish the same tasks they would in a face-to-face consultation, maintaining at least the same quality of care, but they cannot rely on the practices and procedures they would conventionally use. They are thus faced with the challenge of developing methods for completing a physical examination over video in real time. In this paper, we explored the interactional and technological challenges of conducting video examinations and how they are overcome.

## Methods

### Study Design

This paper forms part of the Qualitative Analysis of Remote Consultations study, focused on identifying the communication strategies that make up a *good* video consultation (see protocol for details [21]). Our focus here is on seven video consultations (using FaceTime) between heart failure specialist nurses in Oxford and community-based patients having routine heart failure reviews, including physical examinations (typically measuring weight, blood pressure, heart rate, and rhythm [using a blood pressure monitor put on by the patient or relative and incorporating irregular heartbeat indicator to assess for atrial fibrillation] and oxygen saturation; assessing edema in ankles and legs; and performing chest auscultation for signs of fluid overload or infection). Jugular venous pressure is not generally assessed by heart failure specialist nurses. We combined conversation analysis (CA, an established technique allowing fine-tuned analysis of interaction) [22,23] with ethnography of communication [24] to examine how participants use different modes of communication (eg, speech, gesture, and gaze) in video examinations and why (eg, to compensate for the restricted visual field of the technology), and to gain an understanding of the institutional and situational context in which video examinations take place. Microanalysis of video examinations [25] allowed us to understand how participants decide who speaks when [26], how and when they accomplish actions (eg, instructions and requests for help) [27,28], and how they use these actions to identify and negotiate the challenges of doing video examinations [29].

### Data Collection

Video consultations involved all 5 members of a community heart failure specialist nurse team who were piloting the use of tablet devices for video consultations. All 7 patients had heart failure with reduced ejection fraction (largely a disease of older people, many of whom experience extreme tiredness and multimorbidity), were known to the nurses (having regularly attended follow-up appointments in community clinics), were considered clinically stable, and had sufficient health/digital literacy to participate in a video consultation. As this was a new and potentially risky service model, a doctor-researcher visited each patient at home at the time of the video consultation to troubleshoot the technology, repeat the examination, and check if the patient had any concerns.

We recorded both ends (clinic and patient’s home) of each video consultation, using either small digital camcorders (Sony Handycam DCR-SR72; Sony Corporation) or a handheld iPad (Apple Inc), capturing as much as possible of each individual and their screens as well as contextual details (eg, layout of the room).

### Analysis

Initial exploration of data raised questions about how the technology was being used in video examinations (eg, to observe patients’ legs or ankles), problems experienced when using the technology (eg, limited visual assessment via the technology on the part of clinicians), and changes in participant roles (eg, from clinician to instructor or relative to assessor) [30]. Through this process, we identified three recurrent challenges to conducting video examinations in heart failure reviews: (1) how nurses give instructions to guide patients through video examinations, (2) how nurses and relatives accommodate the patient’s desire for autonomy, and (3) how patients do a physical examination while simultaneously making it visible to the nurse. We focus on these three challenges, as they were relevant for all seven examinations, that is, we found stretches of talk where participants asked and provided clarification (ie, conducted *interactional repair* [31,32]) or there was interactional friction (eg, interruptions [33]). Other challenges were present (eg, cameras in a phone and tablet are very sensitive to overexposure, which, depending on the light, could make assessing edema difficult), but were only relevant to one or two consultations at most.

We transcribed video examinations following CA conventions [34] (Multimedia Appendix 1), allowing us to analyze the details of participants’ talk. We used only limited conventions in the presentation of the data here to maintain legibility. We added screengrabs to illustrate how participants use their bodies (presented in findings using a filter to protect identities). We then built *collections* of all instances of each challenge [23] (157 cases for challenge 1, 18 for challenge 2, and 19 for challenge 3), and analyzed each collection focusing on the verbal and nonverbal communication strategies that participants used when negotiating the challenges of video examinations [35-37].

Our study of video consultations in heart failure received ethics approval from South Central–Berkshire Research Ethics Committee (15/SC/053). All participants consented to anonymized data being used for research, teaching, and reporting.

## Results

### Main Findings

Video examinations were new to all participants. All seven video consultations were successfully completed but involved clinicians and patients working collaboratively to perform examinations and provide results, sometimes with the help of a relative. In three cases, a doctor-researcher provided assistance (once when a blood pressure monitor battery ran out and twice to position the patient’s tablet or laptop to aid examination of edema). The average duration of a video examination was 6.8 min (range 4.7-11.3 min), in consultations of 21 to 48 min.

Below, we focus on three challenges of completing an examination and discuss communication strategies that participants used to negotiate these successfully. Our analysis hones in on successful negotiation of challenges, but that is not to say these video examinations were straightforward. In Multimedia Appendix 2, we provide an extended discussion of one case (Table 1 below) to demonstrate the turn-by-turn challenges of video examinations.

**Table 1:** Example of a patient reporting oxygen saturation readings (data recorded at the patient end).

Line number	Speaker	Turn-at-talk	Screengrabs
01	Patient:	ninety two. (Screengrab 1)	Screengrab 1 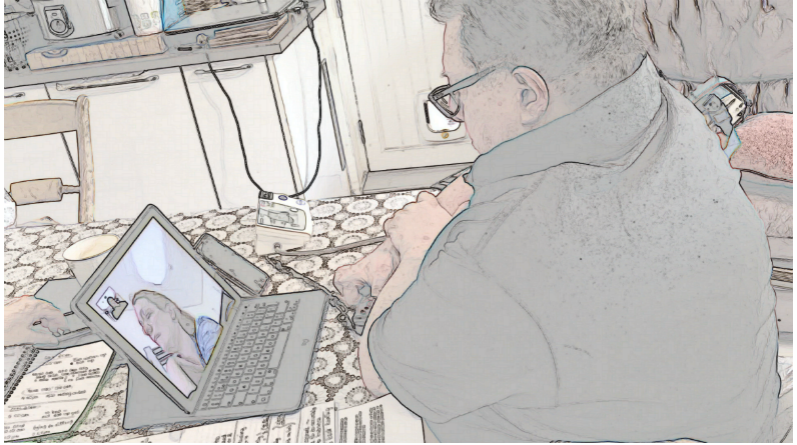
02	Silence	(0.8) (Screengrab 2)	
03	Nurse:	okay. excellent, thank you,	
04	Silence:	(0.2)	
05	Patient:	ninety three.	
06	Silence:	(0.7)	
07	Nurse:	yay. uh[^a^u hu	
08	Patient	[ninety five.	
09	Partner:	uhhu hu	
10	Silence:	(0.5)	Screengrab 2 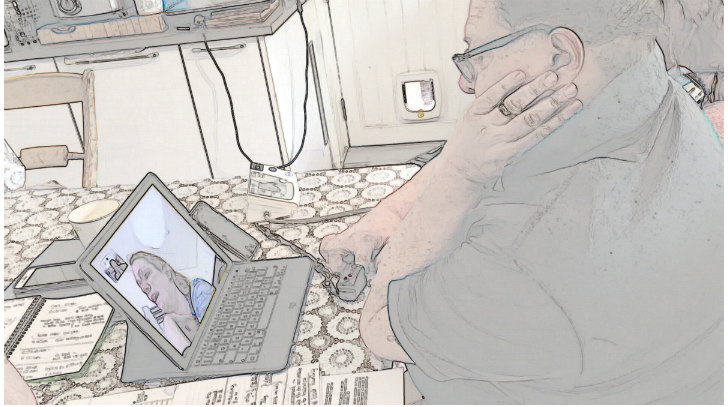
11	Patient:	ninety five;	
12	Silence:	(0.5)	
13	Nurse:	that's great.	
14	Partner:	ninety six;	
15	Patient:	it's ninety six; yeah.	
16	Nurse:	weeh; the dizzy heights;	

^a^Square brackets delineate where participants talk at the same time.

### Challenge 1: How Nurses Give Instructions to Guide Patients Through Video Examinations

Nurses successfully guided all 7 patients through video examinations. Doing so relied on good *recipient design* [26], that is, designing and giving instructions and explanations that accommodated patients’ knowledge about the examination. This involved nurses in the process of assessing patients’ knowledge about what each examination was for (based on their experience of their condition and as a patient) and communicating without jargon. Consider the example in Table 2, in which the nurse needed to know if the patient had an oximeter. She refrained from using the technical term *oximeter* and instead used the descriptive formulation "little oxygen thing" while simultaneously moving her index finger and thumb together and apart repeatedly, depicting how the oximeter is a hinged, crocodile clip–like device opening at one end to enable fingertip insertion (see Table 2, Screengrabs 3 and 4) [38].

**Table 2:** Example of a heart failure specialist nurse explaining the use of an oximeter to a patient (data recorded at the clinic end).

Speaker	Turn-at-talk	Screengrabs
Nurse:	okay. (.) thanks.	Screengrab 3 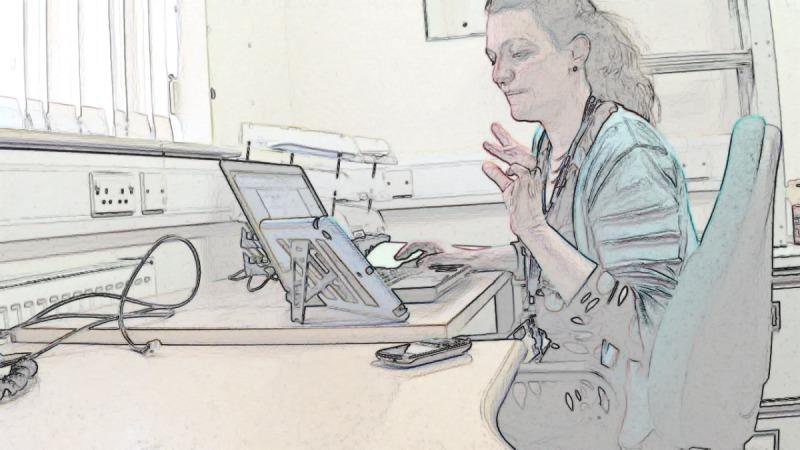
Silence:	(1.3)	
Nurse:	and uhm (0.3) do you have a little oxygen	
Silence:	(0.5) Screengrabs 3 and 4	
Patient:	sats then.	
Nurse:	thing: to go on your (.) finger,	
Silence:	(3.5)	
Patient:	yeah I put (it/that) o:n.	Screengrab 4 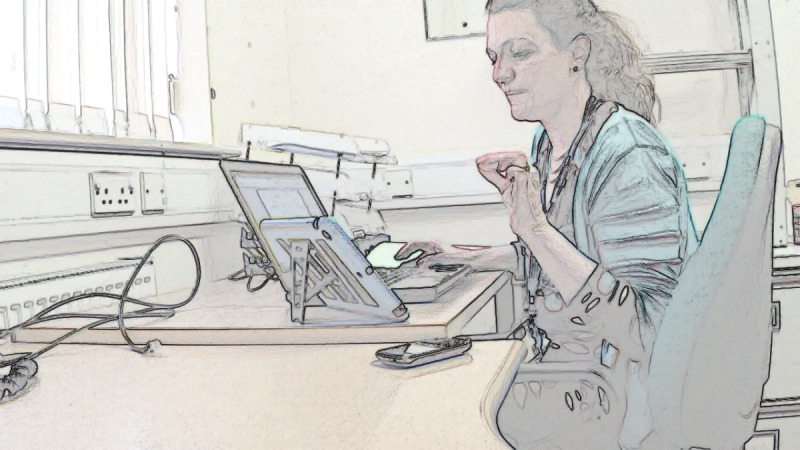

By describing and depicting the oximeter instead of naming it, the nurse treated the patient as someone unfamiliar with the technical name, that is, a nonclinician [39,40]. At the same time, she revealed an assumption that the patient would have knowledge of the oximeter, not by its name but as a "little thing" that is used for oxygen that moves around a hinge and that goes on his finger. In other words, she assumed that he had knowledge of the device based on how it is conventionally used. The patient confirmed that this was an adequately designed explanation by saying "says" (short for oxygen saturation), confirming familiarity with this part of the examination.

This combination of verbal descriptions and visual depictions was used across our dataset and appeared to be key in giving instructions via the video medium (the combination of verbal and nonverbal explanations making optimal use of the visual modality [29]). In the instances when nurses did use technical language such as *oximeter*, they also described the device, held an example up for the camera, or showed how it was used.

Nurses consistently provided upward of 20 instructions in a single consultation. In all seven consultations, patients accepted instructions and explanations and successfully completed the examination. The challenge for nurses was to make correct assumptions about what patients knew to instruct them. These assumptions were not always correct: in eight instructions, nurses assumed that the patient knew more or less than they did. This did not appear to cause issues for patients or relatives who simply sought clarification [31,32].

Our dataset contained one example of when a patient overestimated their own expertise. The extract in Table 1 (an extended transcript and analysis is provided in Multimedia Appendix 2) relates to a patient who had the oximeter on his right index finger, as instructed by the nurse, but the readings he had already provided were low: initially 94% and then 91% (a normal reading is 96%-100%). The nurse instructed him to take some deep breaths, but (line 1) the patient still reported only 92%.

In line 3 (Table 1), the nurse used *okay* to show that she wants to move on with the next step of the consultation [41-43]. She thereby accepted the low measurements as accurate [44]. But then the patient started reporting higher numbers, initially 93 (line 5) and settling on 96 (line 15), which the nurse positively evaluated in lines 7, 13, and 16. After the consultation, the nurse reflected that because the patient initially reported low saturation levels, she was concerned he had pneumonia.

The cause of the low readings was that the patient held his left hand on his right arm (see Table 2, Screengrab 3), thereby limiting the blood flow to his right index finger to which the oximeter was attached. At the point where the nurse had accepted the readings, thus tacitly indicating that this part of the examination had come to an end, he removed his hand (see Table 2, Screengrab 4), restoring normal flow. The patient had thus been conducting the examination incorrectly, without realizing, and leading him to read off low numbers. The solution and correct readings were thus arrived at not by good communicative practice but serendipity. None of this was visible to the nurse via the technology (the tablet camera not being positioned to capture the patients’ arms), who thus could not know that the patient was not performing the examination correctly.

This example illustrates that it is crucial for the clinician to have a clear view of how the patient is performing the examination and that they must not only design instructions to suit the patient’s knowledge and expertise but also monitor how these instructions are carried out.

### Challenge 2: How Nurses and Relatives Accommodate the Patient’s Desire for Autonomy

When patients do their own physical examination in a video consultation, they necessarily have an active role in monitoring and assessing their own body. The video consultation may, therefore, be a good environment to support improved patient autonomy and self-management [45].

In our study, we found that self-examination brought challenges: different patients desired different levels of autonomy (eg, 1 patient found instruction on self-assessment of edema, involving pressing their feet and lower leg to assess for fluid retention, helpful and planned to carry out future self-assessment themselves; others were less enthusiastic), and there was an apparent tension between supporting the patient’s autonomy over their own body and illness and the role of the relative in enabling a video examination.

We identified three cases of patients actively resisting challenges to their autonomy and competence. In the example in Table 3, the patient was in the process of putting on a blood pressure cuff, having told the nurse that the doctor-researcher (present during all video consultations in the study) had already explained how she should take her blood pressure. While trying to put on the cuff, the patient questioned whether it was the right way up (with the inflation tube coming down her arm). At that point, the nurse asked the patient’s relative to help (lines 6-7). Before the nurse could finish the request, the patient interrupted to say she could do it herself (line 8), thereby resisting the call to help.

The nurse asked the relative to help out *before* the patient had a chance to perform the examination. In doing so, she revealed doubts about the patient’s capacity to manage the blood pressure meter and attempted to mobilize the relative to help. The patient interrupted, resisting the challenge to her autonomy and competence. Moreover, the patient confirmed that she is “gonna have a go.” By saying, “give me a moment,” she treated the nurse’s request for the relative to help as coming too soon. As she subsequently explained, she was fine.

Part of the challenge around autonomy relates to the *participation framework* of a consultation (ie, the roles that a clinician, patient, and relative adopts, eg, as an active coparticipant or observer) [46]. Consultations typically involve a clinician and patient. When a relative is present, the nurse can manage the constraints of the mediated setting by changing the participation framework: relatives may be asked to take an active role, supporting and possibly speaking on behalf of the patient, which may have benefits but also risks sidelining or even excluding the patient [47]. Consider the example in Table 4: following an examination of the patient’s oxygen saturation, which the nurse positively evaluated, she wanted to examine the patient’s legs for edema. To self-examine their lower legs, the patient is required to bend over, which can be difficult (sometimes impossible) for patients with heart failure as it can induce breathlessness (*bendopnea*). At this point of the consultation, the patient’s daughter had barely been involved—she was not visible to the clinician, and the interaction had largely been between nurse and patient. However, at the start of the extract, the nurse calls the daughter by name. And when she appears on screen, the nurse informs her of what she wants her to do, essentially bypassing the patient.

**Table 3:** Example of a patient resisting help from a relative during a video examination (data recorded at the patient end).

Line	Speaker	Turn-at-talk
01	Patient:	was it that way or that w- no that way up.
02	Daughter:	are you gonna have a [go?
03	Patient:	[yeah that's right,
04	Silence:	(0.4)
05	Patient:	ye:s, [yeah
06	Nurse:	[((name daughter)),
07	Nurse:	leap in if [you feel she needs (a hand)]
08	Patient:	[g i v e m e a mo]ment
09	Patient:	((name nurse)); I'm very well getting there.

**Table 4:** Example of a nurse involving a relative into a video consultation (data recorded at the patient end).

Speaker	Turn-at-talk	Screengrabs
Nurse:	I'm happy with that,	Screengrab 5 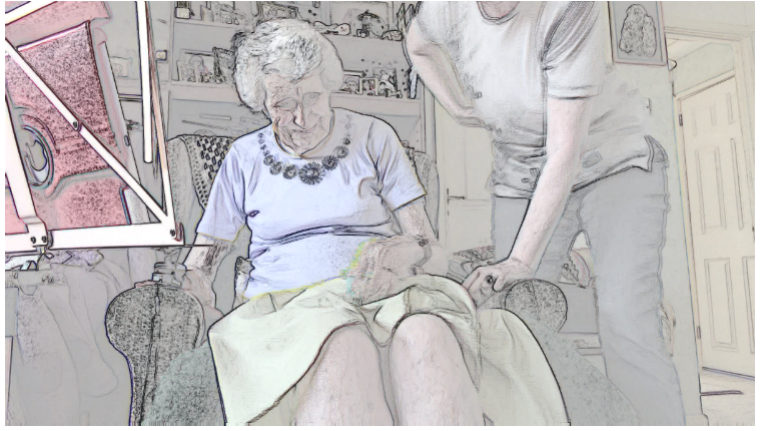
Patient:	Ghoh	
Silence:	(0.7)	
Nurse:	hu hu [hu hu hu	
Daughter:	[oh good,	
Nurse:	darling, (0.3) ((daughter’s name))?	
Daughter:	ye[s? (Screengrab 5)	
Nurse:	[I wanna che- I wanna check (.) your mum's legs; for swelling.	
Daughter:	right, okay, (Screengrab 6)	Screengrab 6 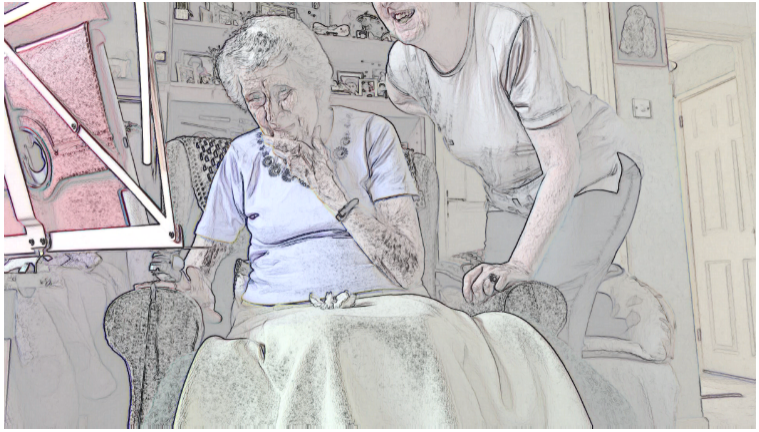

The exclusion of the patient from the *participation framework* is noteworthy. By addressing the daughter and stating that she wanted to check *her mum*, the patient’s role was changed from active coparticipant to *clinical object* [46,48]. Although the severity of her condition prevented her from performing the examination—which the nurse likely knew—earlier interaction indicated that she was cognitively capable of consenting to it (as is usual in face-to-face examinations, in which clinicians either ask for permission [48] or respond to patient presentation of their body for inspection [49]). Whether the change in participation framework is problematic is unclear. In this instance, the patient immediately presented her legs for examination, aligning with the activity that the nurse had initiated and accepting the change in her role. She did, however, interrupt the examination later on (data not shown), saying that her legs were fine, thereby seemingly undermining the necessity of the activity from which she was excluded as an active participant.

Of the 18 cases we identified where the nurse or relative spoke on behalf of a patient or assumed responsibility for an examination (potentially, albeit inadvertently, undermining the patient’s competence or autonomy), 15 were not explicitly challenged by the patient. We did find that patients have alternative ways of resisting their exclusion from the interaction, as with the patient in Table 4 who said she was fine while the nurse and relative are conducting the examination.

### Challenge 3: How Patients Do a Physical Examination While Simultaneously Making It Visible to the Clinician

The third challenge we identified was related to how nurses observed and evaluated video examinations. One way was for the patient or relative to tell the clinician what they saw or felt. Verbal communication of some aspect of a physical examination (eg, reading blood pressure measurements from a digital display) was largely unproblematic. Examinations involving physical observation and/or manipulation of a patient’s body were more problematic, with patients and relatives at times struggling to make bodies visible and nurses struggling to observe and assess.

Patients and relatives do not have *professional vision* [50] (ie, they do not have the clinical training that allows them to see and interpret results of examinations). They, therefore, needed to perform physical examinations while, at the same time, making them visible to the nurse. This was challenging for two reasons. First, the patient or relative had to work out how to make the examination adequately visible to the nurse via the technology. Second, the patient or relative then needed to maintain that visual field while performing the examination, meaning they had to attend to the patient’s body and the technology simultaneously. Success was dependent on the type of technology (phone, tablet, or laptop), the presence of a third party who could assist the patient, the patient’s mobility, and the technological expertise of all parties.

The main obstacle patients and relatives encountered when attempting to make the examination visible for the nurse was determining what the nurse could see. Consider the example in Table 5 in which the nurse gives instructions to the patient to assess for oxygen saturation. The patient then aimed his phone at his leg (Table 5, Screengrab 7) and, as a result, could no longer see the video preview on his phone that would allow him to monitor what the nurse can see.

**Table 5:** Example of a patient reporting oxygen saturation during a video examination (data recorded at the patient end).

Speaker	Turn-at-Talk	Screengrabs
Nurse:	would you be able to rest it on the floor.	Screengrab 7 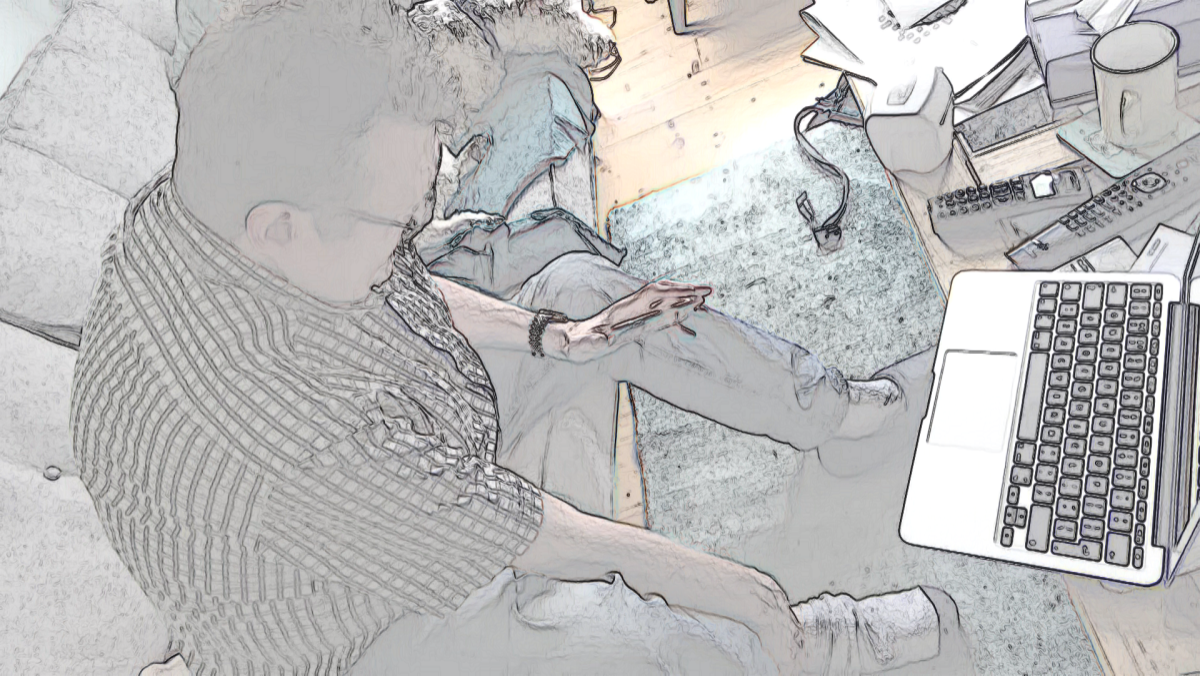
Silence:	(1.6)	
Nurse:	rest it on the floor and then uhm, (0.3) and then give it a little (.) press.	
Nurse:	(0.6) uhm at the (0.6) starting at the bottom,	
Silence:	(1.8)	
Patient:	.h can you see that (Screengrab 7)	
Silence:	(0.7)	
Nurse:	uhmmmm (0.4) just. yes.	
Silence:	(4.1)	
Patient:	any better? (Screengrab 8)	Screengrab 8 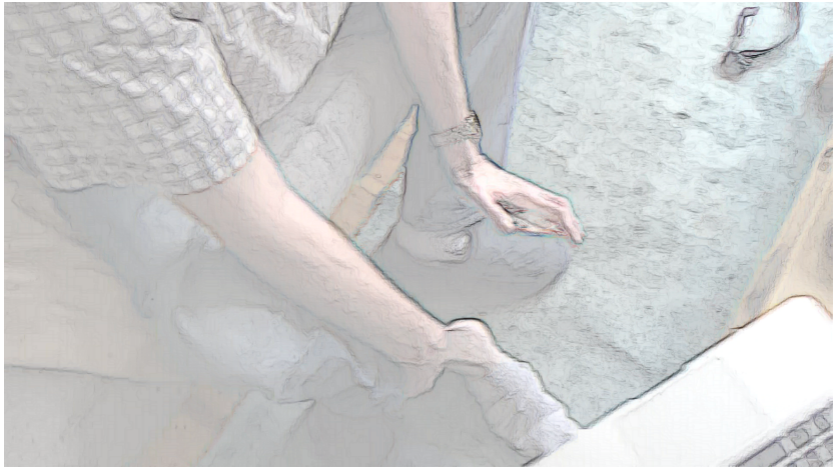
Silence:	(0.5)	
Nurse:	yeah that's good,	

When the patient initially started pressing his leg, he held the phone perpendicular to the floor at knee height. He then turned the camera downward (see Table 5, Screengrab 7) and asked the nurse if she could see (the problem being that the patient could not see what the nurse could see and so had to rely on her feedback). The nurse’s response was delayed—with a silence of 0.7 seconds, a lengthy "uhm", followed by another silence of 0.4 seconds—all indicating she was struggling to give a straightforward answer [51-53]. Although the nurse then confirmed with "yes", she mitigated her answer with "just". The patient subsequently moved the phone closer to his ankle (see Table 5, Screengrab 8), indicating that he had understood that she could not adequately see and then asks if it was “any better”. This time the nurse not only confirmed but also gave a positive evaluation. She then resumed the examination.

This analysis exemplifies the challenge of providing visual access: the use of video technology means that patients cannot always see if they are showing their body correctly to the clinician at the other end [54,55]. To make the examination visible, the patient or relative needs to aim the camera (also the screen they use to monitor the nurse’s field of vision). The result is a complex collaborative arrangement involving the patient (who cannot see if the examination is visible to the clinician), the clinician (who needs to give instructions and feedback to enable visual assessment), and the technology (which needs manipulating at the patient’s end to enable an effective video examination).

**Figure 1:**
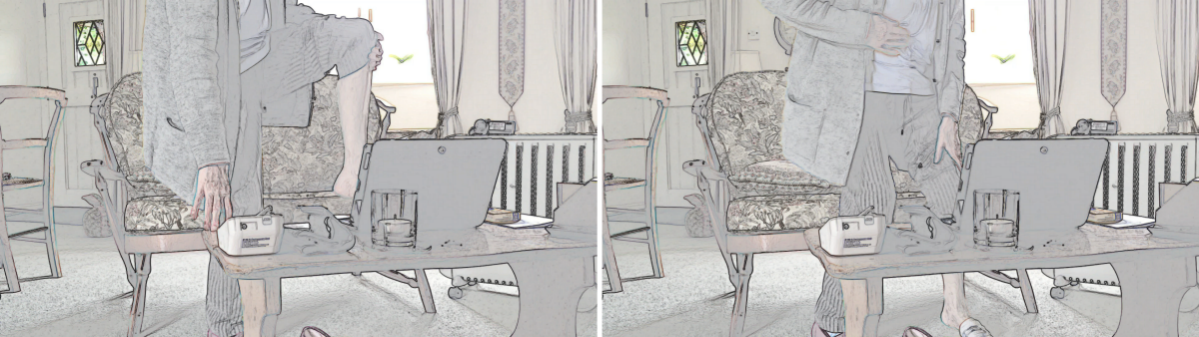
Patient attempting video examination whilst standing.

Once a clinician has visual access, they need to maintain it. We identified five cases where this went well: patients had no mobility problems (as in Table 5) or relatives made effective use of the *affordances* of the technology (ie, the actions made possible by an object in a particular setting [29]); for example, holding the tablet while the patient (who could then see the screen) performed the examination and instructed them how to aim the camera. In two cases, both with patients with limited mobility, maintaining visual access on the part of the nurse proved difficult. Take the screengrabs in Figure 1 in which a patient initially managed to provide the nurse with visual access to her leg before experiencing a cramp and lowering her leg back to the floor (thereby losing visual access for the nurse). The patient then sat down before pressing her leg to test for edema, leaving the nurse to rely on the patient’s verbal confirmation—combined with the patient’s later assertion that she had lost weight—that she did not have edema.

## Discussion

### Principal Findings

Our findings demonstrate that physical examinations in video consultations are sometimes possible but are not straightforward replacements of in-clinic examinations. The combination of multimodal recordings of video consultations with microlevel analysis of video examinations, using an established, systematic approach, has allowed us to do the following: First, we have shown that accomplishing video examinations involves a collaborative process with patients, clinicians, and (sometimes) relatives. In the context of heart failure services, this involves rethinking the interactions that typically take place in a face-to-face clinical consultation. Second, we have shown how video examinations are inherently shaped by technology in use. Patients and those supporting them need to understand and manipulate the technology to enable observation and evaluation on the part of the clinician. This involves a complex process of giving and receiving instructions, manipulating technology (the affordances of which can subtly impact the examinations), and ensuring visual presence. This combination can be physically challenging (particularly for older people with limited mobility), practically tricky, and time consuming. For heart failure specialist nurses, it also involves an awareness (built up over time) of patients’ knowledge and experience of their condition, the technology, and the requirements of physical examination. Third, we have highlighted the potential of video examinations to extend patient autonomy and self-management. The lack of physical copresence and the use of video medium requires patients (and sometimes relatives) to take an active role in assessment. Some patients appear to value video examinations as an opportunity to learn how to do self-assessment and manage their own condition. However, caution is needed as some patients may overestimate their expertise, potentially leading to incorrect assessment (and inaccurate results).

In sum, our data suggest that participants in heart failure consultations develop new communication practices that enable them to successfully negotiate the interactional and technological challenges of video examinations. This confirms that, at least in some cases, video examinations are feasible.

### Comparison With Previous Research

Research on video consultations typically focuses on the feasibility and acceptability of video technology and allied services, with limited appreciation of video examinations. To date, only one study has been published focusing on video examinations, but patients were collocated with a nurse who assisted the specialist with video examination [19]. Evidence from outside of health care indicates subtle changes in interactions when using video conferencing (and hence, eg, potential for misunderstandings) [56-58]; but in health care studies, we have yet to examine implications of video-mediated interactions (focusing instead, for instance, on how video consultations get started [59,60], how participants show engagement, and the effective use of objects [18]).

Our study, therefore, offers a small but important contribution. To our knowledge, it is the first study to focus specifically on video examinations. As such, we confirm previous work (focused on phone consultations, refer to the study by Lopriore et al [17]) that the lack of a shared physical environment poses new challenges for clinicians and patients. We have built on this by demonstrating that remote physical consultations are possible via video and that they involve a collaborative, sociotechnical process. We have identified three key challenges to video examinations and potential means of addressing them. Patients do not necessarily need assistance from a copresent health care provider to perform a video examination, but they do appear to need clear instructions and guidance from clinicians (at the other end), a solid appreciation of the technology and examination, and (sometimes, but not always) support from relatives particularly when simultaneously manipulating body and technology. Our findings also add to broader work on video interaction by demonstrating how challenges characteristic of video-meditated interaction [10,57,61] are relevant in health care settings and can be collaboratively negotiated.

Previous studies have shown that lack of appreciation of patients’ desired autonomy on the part of the clinician, combined with a focus on relatives over patients, can be detrimental to patient engagement, self-management, and quality of care [47]. We have shown that in video consultations, clinicians can guide some patients to take responsibility for their own examination and potentially enhance autonomy. This appears relevant to patients with heart failure (and possibly other long-term conditions), who are often experts in their own condition, have experience with the relevant procedures, and established relationships with the clinical team [62].

### Strengths and Limitations

This was an exploratory study, drawing on a small sample of video-recorded consultations in a single heart failure service. Data have allowed us to examine whether video examinations are possible, and our microanalytic approach has enabled us to identify key challenges experienced by clinicians, patients, and relatives as well as strategies for potentially overcoming them. Our methodology is transferable to the study of physical examinations in other clinical conditions and settings.

However, there are clear limits to the transferability of our findings. Our focus on patients with heart failure meant that we examined the use of video examinations with a group that are typically older, dependent, and have limited mobility (often because of breathlessness associated with the condition) and multimorbidity. Many struggled with the physical, practical, and technological challenges of video examinations and needed help from a relative to successfully complete the examination. It is likely that a sample of patients with a different condition (eg, type 1 diabetes) would not have the same struggles. Younger patients might have a particular aptitude. Further research is needed to appreciate whether video examinations might be less challenging with those experiencing other conditions.

Recordings were made early in the piloting of a remote consulting service. This meant that clinicians, patients, and relatives received no training or preparation for conducting a video examination. Given the complex collaborative process involved in performing video examinations and the need for clear instructions (that likely differ from those in face-to-face consultations), those replicating or extending this work are advised to build in adequate training and support, particularly for those new to the video medium.

### Conclusions

It is sometimes possible to conduct a physical examination in a video consultation. Video examinations appear feasible for some patients with heart failure, some of the time, but there are significant interactional and technological challenges for all involved. Clinicians and patients require sound appreciation of the technology involved and need to work together to perform video examinations. Further research is needed to understand if other patients with other conditions would find video examinations less challenging. Developers in this space need to work with providers to consider how their devices/software can facilitate video examination. Decision makers would do well to appreciate the challenges of video examinations and the time involved (in setting up as well as doing). Given patient and clinician caution around video examinations and the challenges participants encounter in developing new ways of working, guidance and training are urgently needed to support patients and clinicians in gaining the appropriate experience, knowledge, and interactional skills necessary to successfully manage video examinations. Without this, widespread uptake of video consultations is unlikely.

## Appendix

Multimedia Appendix 1 Transcription conventions.

Multimedia Appendix 2 Extended analysis of example in Table 1.

## Abbreviations

CA: conversation analysis
NIHR: National Institute for Health Research
OTQS: Oxford Telehealth Qualitative Study
