# Supplementary material for: Physical Examinations via Video for Patients With Heart Failure: Qualitative Study Using Conversation Analysis
Source: J Med Internet Res. 2020 Feb 20;22(2):e16694. doi: 10.2196/16694 (PMC7059096; doi:10.2196/16694)
Supplement: Multimedia Appendix 1 [file jmir_v22i2e16694_app1.docx]

| *Transcription conventions (Jefferson, 2004)* | |
| --- | --- |
| Symbol | Meaning |
| . ; _ , ¿ ? | Turn-final strongly falling, medium falling, flat, slightly rising, medium rising, or strongly rising intonation |
| (.) | Hearable “micropause” of less than 200ms |
| (1.0) | Hearable silence of 1s |
| Cu- | Hyphens indicate a hearable cut-off |
| [Talk] | Square brackets enclose overlapping talk |
| (Talk) | Transcriber unsure of accurate transcription |
| .hhh | Hearable inbreaths |
| Hhh | Hearable outbreaths |
