# Supplementary material for: Physical Examinations via Video for Patients With Heart Failure: Qualitative Study Using Conversation Analysis
Source: J Med Internet Res. 2020 Feb 20;22(2):e16694. doi: 10.2196/16694 (PMC7059096; doi:10.2196/16694)
Supplement: Multimedia Appendix 2 [file jmir_v22i2e16694_app2.docx]

**Multimedia Appendix 2 – Extended analysis of example in table 2**

In the *Results* section we showed an example of a patient overestimating their own expertise (Table 2). The patient had to report their oxygen saturation using an oximeter. While he managed to report the numbers, he incorrectly performed the examination, resulting in him reporting low numbers. This caused the nurse concern about whether he has pneumonia.

Here we provide a more extensive analysis of the same data. First, we use this analysis to demonstrate that while the challenge is resolved in the end, might could suggest it is not in fact a challenge, this process was not straightforward. Second, it shows that while we provided few details for the analyses in the main manuscriptd, our findings are the result of an in-depth analysis of the interactional data. We include a complete transcript starting when the nurse asked the patient to put on the oximeter (also shown in Table 1 in the main manuscript) and ending when the patient took the oximeter off his finger.

**Table 2a – Example of a patient reporting oxygen saturation readings***

| 01 Nurse: okay. (.) thanks:.  02 (1.3)  03 a:nd u:hm (0.3) do you have a little o:xygen  04 (0.5)  05 Patient: sats then.=  06 Nurse: =thing: to go on your (.) finger,  07 (3.5)  08 Patient: yeah I put (it/that) o:n. #figure10  09 (10.4) #figure11  10 ninety, (0.7) f:ou:r.  11 (1.7)  12 twenty eight, it says, and then a (0.2) hold  13 on, (4.4) (no) sorry,  14 (0.5)  15 Nurse: (right),  16 (12.8) | |
| --- | --- |
| 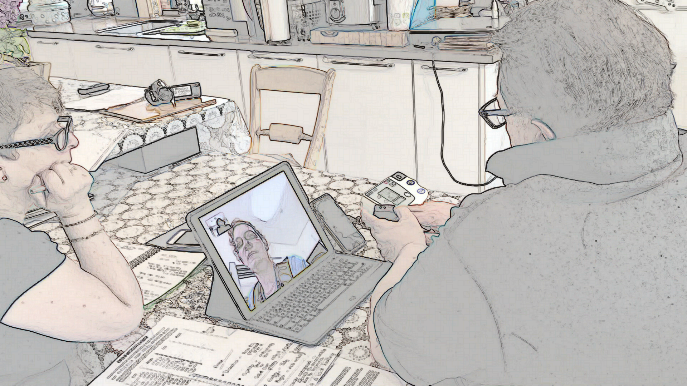  Figure 10 – Patient puts on oximeter | 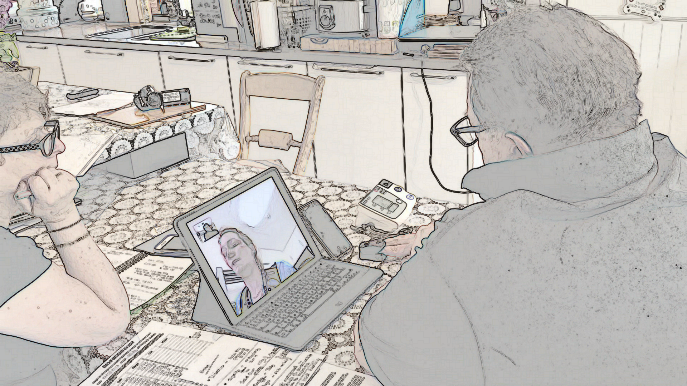  Figure 11 – Patient puts left hand on right arm |

* Data was recorded at the ‘patient end’ of the consultation

The patient put the oximeter on his finger in line 7 and then informed the nurse that he had done so (see figure 10). The next step would be to provide her the numbers on the oximeter: one represented his heartrate, the other his oxygen saturation. This already proved problematic, because the patient id not know what each number represented. He initially in line 10 provided an oxygen reading of ninety-four, but then in line 12 provided a number that could not be correct: twenty-eight. He broke off this utterance and after some silence stated that he did not know which of the numbers he should have been reporting. Note also that at this point he had already put his left hand on his right arm (see figure 11), obstructing the blood flow and starting a problem that would contribute to the examination taking longer than it needed have.

**Table 2b – Example of a patient reporting oxygen saturation readings***

| 17 Patient: I don't know which number I'm looking at.  18 (0.7)  19 Nurse: u:hm do you wanna show me on the (.) FaceT[ime;=  20 Partner: [i-  21 it's:- (.) it's this way. (0.2) isn't it.  22 (1.1) #figure12  23 Patient: it's facing you:, #figure13  24 Partner: yeah. | |
| --- | --- |
| 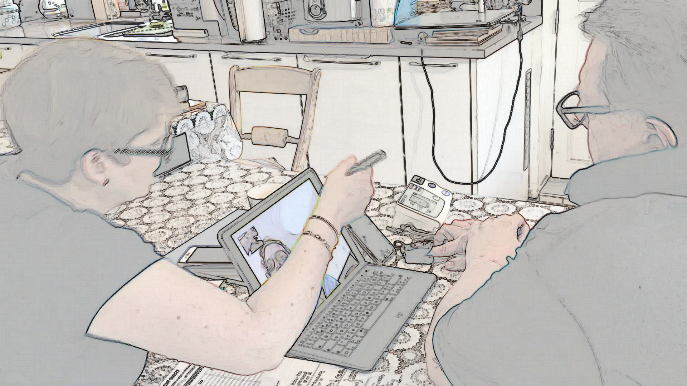  Figure 12 – Partner indicating how to read oximeter | 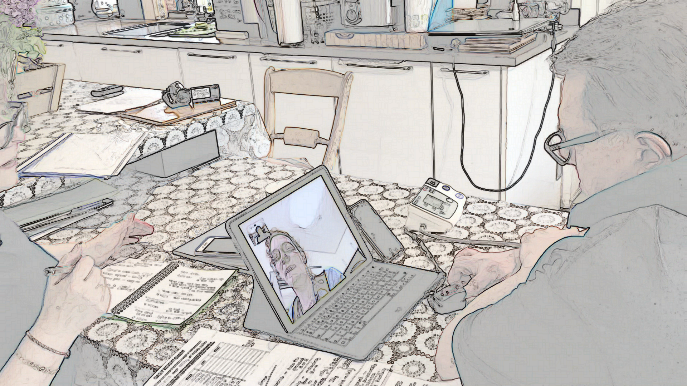  Figure 13 – Patient shifting posture |

* Data was recorded at the ‘patient end’ of the consultation

Once the patient had stated his uncertainty, the nurse provided a solution: she requested in line 19 for him to hold the oximeter to the camera. But in overlap the patient’s partner offered a candidate explanation of how the patient should have read the numbers. And instead of holding the oximeter up for the nurse, both she and the patient began reading off the numbers again starting in line 26. Had the patient moved the oximeter, he would necessarily also have removed his left hand, but instead he just slightly shifted his posture, maintaining his hand positions and obstructing the blood flow (see figures 12 and 13).

**Table 2c – Example of a patient reporting oxygen saturation readings***

| 25 (2.6)  26 it's ninety over seventy s:::even.=  27 Patient: ninety,  28 (4.1)  29 Partner: °ninety one over [seventy (seven)  30 Patient: [ninety on:e,  31 (0.4)  32 Partner: it keeps ↑cha:nging,  33 (1.4)  34 Nurse: does it (0.2) flash or: u:hm  35 Patient: it's flashing ↑green now.  36 (2.4)  37 over seventy fi:ve?  38 (0.8)  39 °se[venty fou:r°, |
| --- |

* Data was recorded at the ‘patient end’ of the consultation

The numbers did not stabilize however, and the partner reported this in line 32. The nurse again offered help, asking if it was flashing, which would have indicated that the oximeter had gotten a reading. The patient confirmed that it was green, and again reported a number. Note that both the patient and his partner formulated the numbers as one *over* another, the way one would report blood pressure results. For example in line 26 the partner said ninety of seventy-seven. They thereby showed, unintentionally, that they lacked a complete grasp of what the numbers meant.

**Table 2d – Example of a patient reporting oxygen saturation readings***

| 40 Nurse: [wha- the- is the other number the ninety one;=  41 Patient: =nine- (.) ninety t:wo: that is at the top,  42 (2.7)  43 and then (there's:) one underneath it:, (.)  44 is seventy three.  45 (0.5)  46 Nurse: [okay. take some dee- (0.3) deep breaths for me¿  47 Patient: [(is that all-)  48 ((patient breathes in and out twice))  49 Nurse: okay just breathe normally¿  50 (6.4)  51 Patient: .mlk.h °yeah it's going up°.  52 (1.7)  53 .mlk.h now it's n- ninety two:.  54 (5.7)  55 ninety one;  56 (15.6)  57 ninety two.  58 (0.8)  59 Nurse: okay.=excellent,=tha:nk you:, |
| --- |

* Data was recorded at the ‘patient end’ of the consultation

After the nurse had asked for clarification on the numbers (line 40), the patient read out both. This indicated to the clinician what his heartrate was and that his oxygen saturation was low. But she did not inform the patient and instead asked him to breathe deeply. This may have suggested that there was a problem, and possibly the nature, but not its specifics. After the patient had taken two breaths, he reported that the number was going up, using *yeah* to confirm that the solution offered by the nurse was working. But the numbers remained low: around ninety-one, ninety-two. The nurse finally accepted these numbers in line 59, using *okay*, *excellent*, and *thank you*, as indicators that she was ready to move on to the next part of the consultation. She thus took the ninety-one or ninety-two as the official readings.

At this point the patient had had his right hand on his left arm during the entire procedure, limiting the flow of blood to the finger on which he had attached the oximeter. But none of the participants had noticed this and thus none were aware that the readings were likely low simply because the patient had been conducting the measurement incorrectly. Once the examination had come to completion, the patient removed his right hand, restoring the blood flow, and causing the readings to rise rapidly. In the end they settled on ninety-six. A far more comforting number.

**Table 2e – Example of a patient reporting oxygen saturation readings***

| 60 (0.2)  61 Patient: .mlk ninety three:.  62 (0.7)  63 Nurse: £yea:£. uh[:u hu  64 Patient: [ninety fi:ve.  65 Partner: uh::hu hu  66 (0.5)  67 Patient: ↑ninety fi:ve;  68 (0.5)  69 Nurse: that's great.=  70 Partner: =↑ninety six;=  71 Patient: =it's ninety six; yeah.  72 Nurse: ↑wee::h; the dizzy heights; |
| --- |

* Data was recorded at the ‘patient end’ of the consultation

This extended analysis shows that an examination that may seem straightforward, can in practice be challenging. The patient should merely have had to put the oximeter on his finger and read out the numbers or hold the oximeter up to the camera for the nurse to read. Instead it took slightly over 2.5 minutes for the examination to be completed. Part of the problem was that the patient was unwittingly causing the oximeter to provide low numbers. He performed the procedure incorrectly, which leas to the nurse asking him to breathe deeply, and to wait a while before finally accepting the number he reported.

That the examination was completed successfully was also not a result of good communication practices, but came about serendipitously. The patient just happened to remove his hand while he could still read out the oximeter readings. Had he taken off the clip immediately, they would not have known that he had been providing incorrect readings. It is interesting to note that the nurse had already offered a solution, but that she refrained from doing so again when it was not taken up by the patient. Had she pursued that course of action, it is likely the examination would have been completed faster and they would have gotten the right results. This shows that while patients and clinicians may get the right result, to avoid this being simply by good luck they require adequate guidance and training..
